# Supplementary material for: Differentially Expressed Somatostatin (SST) and Its Receptors (SST1-5) in Sporadic Colorectal Cancer and Normal Colorectal Mucosa
Source: Cancers (Basel). 2024 Oct 24;16(21):3584. doi: 10.3390/cancers16213584 (PMC11545382; doi:10.3390/cancers16213584)
Supplement: Supplementary file 1 [file cancers-16-03584-s001.zip › Table S1.pdf]

Supplementary Materials:

Table S1. Sequences of primers used for RT-qPCR assay.

Target	Primer sequence	ENST number http://www.ensembl.org	Amplicon length
**SST**	CCA GAC TCC GTC AGT TTC TGC A (F)<br>TTC CAG GGC ATC ATT CTC CGT C (R)	00000157005	122 bp
**SST1**	CGC TCT TTC CAA CGC ATC CTA TG (F)<br>GGA AGT CTT CCA CAC TGT AGG C (R)	00000139874	112 bp
**SST2**	CAA TGG CTC TGT GGT GTC AAC C (F)<br>CTT GGC ATA GCG GAG GAT GAC A (R)	00000180616	157 bp
**SST3**	ATG AGC ACC TGC CAC ATG CAG T (F)<br>CTT CAC CAC GAT GAG CAG GTA G (R)	00000278195	135 bp
**SST4**	CTA TGG CTT CCT CTC CGA CAA C (F)<br>GCT CTT GAG AGC AGT GGC ATA G (R)	00000132671	130 bp
**SST5**	CTT CTT CGT GGT CAT CCT CTC C (F)<br>TTG CGG AGG CAC AGA ACC TTC T (R)	00000162009	111 bp
GADPH	GAA GGT GAA GGT CGG AGT CA (F)<br>GAC AAG CTT CCC GTT CTC AG (R)	00000229239	199 bp
HPRT1	CTG AGG ATT TGG AAA GGG TG (F)<br>AAT CCA GCA GGT CAG CAA AC (R)	00000298556	156 bp

Descriptions: F: forward primer sequence (5′ > 3′), GAPDH: glyceraldehyde-3-phosphate dehydrogenase, HPRT1: hypoxanthine phosphoribosyltransferase 1, R: reverse primer sequence (5′ > 3′), RT-qPCR: real-time quantitative PCR, SST: somatostatin, SST1-5: somatostatin receptors 1-5.
