# Supplementary material for: Differentially Expressed Somatostatin (SST) and Its Receptors (SST1-5) in Sporadic Colorectal Cancer and Normal Colorectal Mucosa
Source: Cancers (Basel). 2024 Oct 24;16(21):3584. doi: 10.3390/cancers16213584 (PMC11545382; doi:10.3390/cancers16213584)
Supplement: Supplementary file 1 [file cancers-16-03584-s001.zip › Table S2.pdf]

**Supplementary Materials:**

**Table S2**. Comparative IHC tissue expression of SST1-5 in colorectal cancer (CRC), lymph node metastasis (LNM) and control colorectal mucosa (C).

| Group | Receptor type | n | Area Fraction [%] | | | | | | | $p^a$ |
|---|---|---|---|---|---|---|---|---|---|---|
| | | | Mean | Median | Min | Max | Q1 | Q3 | SD | |
| **CRC** | **SST1** | 59 | 31.99 | 30.21 | 2.03 | 70.79 | 16.32 | 47.50 | 18.07 | < 0.0001 |
| | **SST2** | 58 | 28.69 | 28.43 | 0.00 | 74.81 | 12.31 | 41.13 | 18.63 | |
| | **SST3** | 61 | 16.35 | 9.06 | 000 | 78.99 | 1.9 | 28.42 | 18.92 | |
| | **SST4** | 59 | 13.97 | 11.78 | 0.00 | 72.44 | 4.12 | 21.13 | 13.51 | |
| | **SST5** | 60 | 36.21 | 36.48 | 122 | 84.23 | 18.36 | 55.19 | 21.79 | |
| **LNM** | **SST1** | 32 | 36.57 | 38.73 | 550 | 67.64 | 24.79 | 46.74 | 17.93 | 0.0003 |
| | **SST2** | 32 | 30.63 | 33.25 | 3.51 | 66.54 | 12.83 | 43.76 | 19.60 | |
| | **SST3** | 31 | 16.58 | 12.34 | 0.00 | 69.22 | 4.54 | 24.93 | 16.42 | |
| | **SST4** | 32 | 23.75 | 25.24 | 1.33 | 52,0 | 10.60 | 32.70 | 14.69 | |
| | **SST5** | 31 | 27.45 | 20.92 | 1.02 | 8030 | 13.44 | 41.33 | 19.53 | |
| **C** | **SST1** | 58 | 19.89 | 16.71 | 0.18 | 53.89 | 8.88 | 29.79 | 14.31 | < 0.0001 |
| | **SST2** | 60 | 11.93 | 9.91 | 0.00 | 42.51 | 3.95 | 17.21 | 9.79 | |
| | **SST3** | 61 | 10.13 | 7.85 | 0.00 | 40.14 | 2.96 | 14.21 | 9.26 | |
| | **SST4** | 58 | 12.61 | 9.74 | 0.00 | 54.91 | 2.79 | 18.55 | 12.44 | |
| | **SST5** | 59 | 17.56 | 15.15 | 0.00 | 47.62 | 5.10 | 28.67 | 13.00 | |

Descriptions: [a]Friedman's rank test and Bonferroni post-hoc test, max: maximum, min: minimum, n: number, SD: standard deviation, SST1-5: somatostatin receptors 1-5, Q1: lower quartile, Q3: upper quartile.
