# Supplementary material for: Differentially Expressed Somatostatin (SST) and Its Receptors (SST1-5) in Sporadic Colorectal Cancer and Normal Colorectal Mucosa
Source: Cancers (Basel). 2024 Oct 24;16(21):3584. doi: 10.3390/cancers16213584 (PMC11545382; doi:10.3390/cancers16213584)
Supplement: Supplementary file 1 [file cancers-16-03584-s001.zip › Table S3.pdf]

Supplementary Materials:

Table S3. Values of Spearman's coefficient for correlation between SST and SST1-5 immunoexpression in colorectal cancer (CRC), control colorectal mucosa (C), age of the patient, and basic laboratory tests.

Type of peptide	Group	Age (yrs)	Glucose (mg/dL)	WBC (x10^9/L)	Total protein (g/dL)
**SST**	**CRC**	-0.16	-0.02	0.80	0.14
	**C**	-0.16	0.25	0.07	-0.50
**SST1**	**CRC**	-0.14	-0.09	0.21	-0.80
	**C**	-0.24	-0.09	0.27	-1.00
**SST2**	**CRC**	-0.13	0.33	-0.11	0.20
	**C**	**-0.45**	-0.07	-0.07	-0.50
**SST3**	**CRC**	**-0.17**	-0.10	0.09	1.00
	**C**	**-0.34**	-0.21	0.34	0.50
**SST4**	**CRC**	**-0.21**	0.16	0.18	0.40
	**C**	**-0.32**	-0.04	0.17	-0.50
**SST5**	**CRC**	0.04	0.13	-0.01	-0.50
	**C**	-0.13	-0.08	0.18	-0.50

Descriptions: bold numbers indicate values of significant R coefficient ($p < 0.05$), WBC: white blood cells.
