# Supplementary figures and images for: Differentially Expressed Somatostatin (SST) and Its Receptors (SST1-5) in Sporadic Colorectal Cancer and Normal Colorectal Mucosa
Source: Cancers (Basel). 2024 Oct 24;16(21):3584. doi: 10.3390/cancers16213584 (PMC11545382; doi:10.3390/cancers16213584)

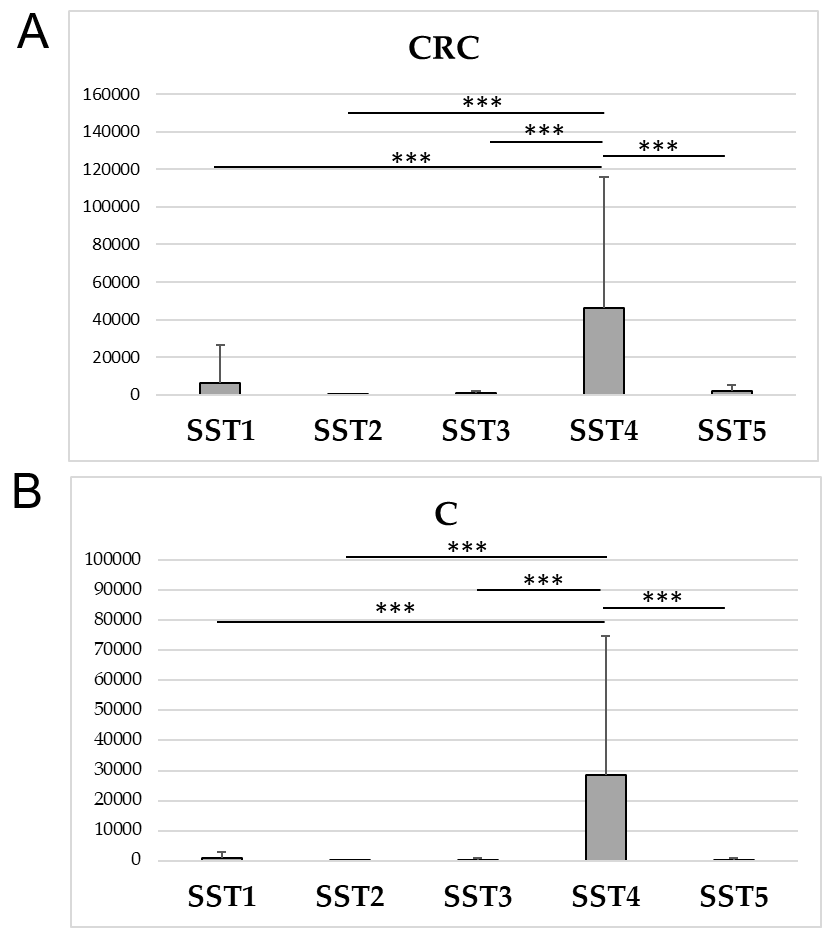

Supplement: Supplementary file 1 [file cancers-16-03584-s001.zip › Figure S1..tif]

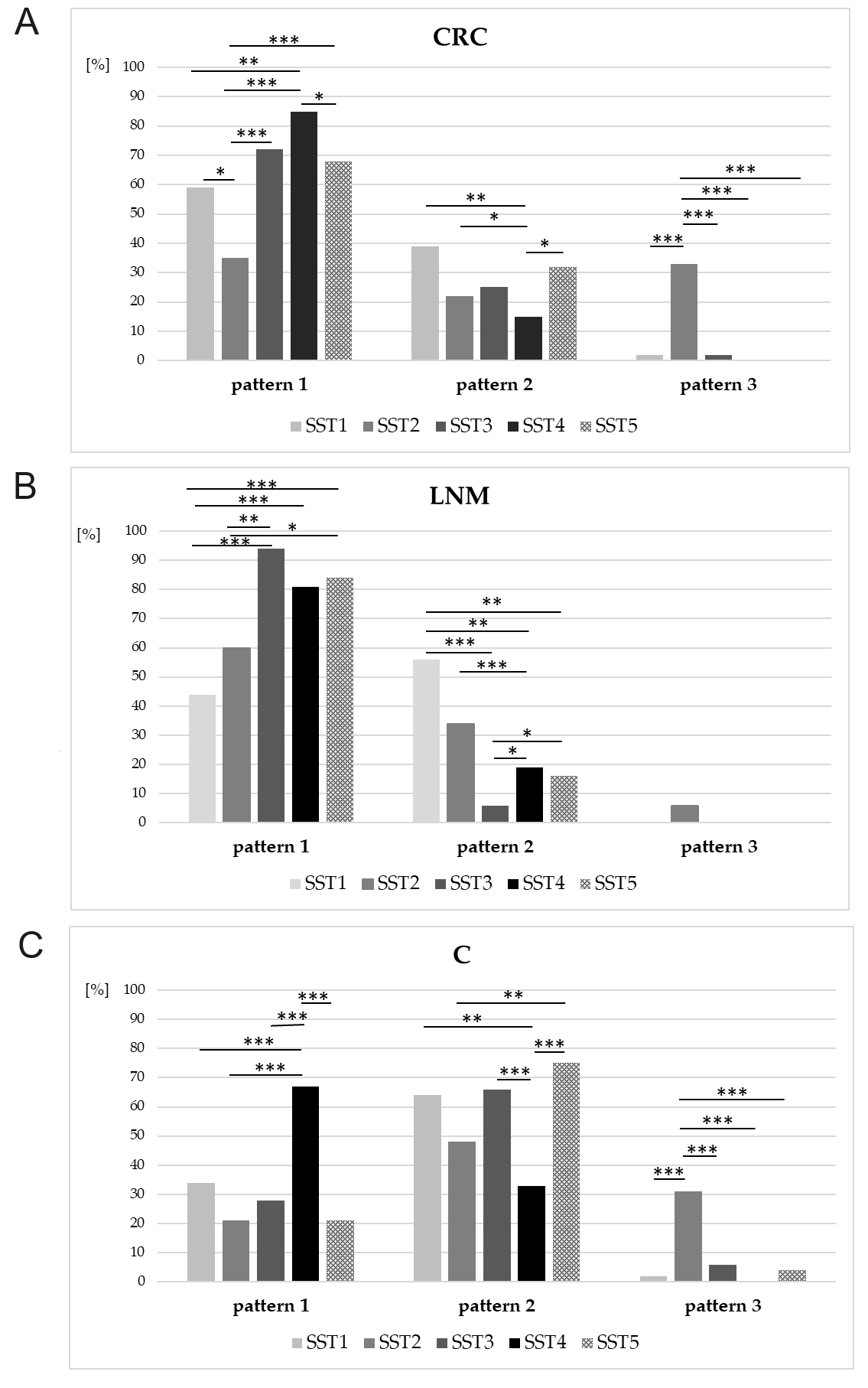

Supplement: Supplementary file 1 [file cancers-16-03584-s001.zip › Figure S2..tif]
